# Piles

**Published:** 1883-05

**Authors:** 


PILES.—We venture to say that at least sixty adults out of every hundred are more or less troubled with piles ; and we further give it as our opinion that in fifty-nine cases out of every sixty the trouble has been caused by using in water closets newspapers, instead of brown tissue paper. Not only is the ink with which white paper is printed an irritant, but the paper itself is far worse, as it contains chloride of lime, which is used for bleaching it.

This fact should be generally known. It would save much suffering to the multitudes who now suffer from this distressing complaint.

